# Nociception-Induced Changes in Electroencephalographic Activity and FOS Protein Expression in Piglets Undergoing Castration under Isoflurane Anaesthesia

**DOI:** 10.3390/ani12182309

**Published:** 2022-09-06

**Authors:** Judith Reiser, Matthias Kreuzer, Julia Werner, Anna M. Saller, Johannes Fischer, Steffanie Senf, Pauline Deffner, Nora Abendschön, Tanja Groll, Andrea Grott, Regina Miller, Shana Bergmann, Michael H. Erhard, Mathias Ritzmann, Susanne Zöls, Gerhard Schneider, Katja Steiger, Christine Baumgartner

**Affiliations:** 1 Center of Preclinical Research, Technical University of Munich, 81675 Munich, Germany; 2 Department of Anesthesiology and Critical Care, School of Medicine, Technical University of Munich, 81675 Munich, Germany; 3 Clinic for Swine, Center for Clinical Veterinary Medicine, LMU Munich, 85764 Oberschleissheim, Germany; 4 Institute of Pathology, School of Medicine, Technical University of Munich, 81675 Munich, Germany; 5 Chair of Animal Welfare, Ethology, Animal Hygiene and Husbandry, LMU Munich, 80539 Munich, Germany

**Keywords:** EEG, FOS, piglet, electroencephalography, castration, local anaesthesia, nociception, lidocaine, procaine, mepivacaine, bupivacaine, spectrogram, animal

## Abstract

**Simple Summary:**

Efficient analgesia during surgical piglet castration is an important animal welfare issue. The present study is part of a larger study designed to investigate the efficacy of four local anaesthetics (bupivacaine, lidocaine, mepivacaine and procaine) for analgesia during castration. In conscious piglets, it is difficult to distinguish between stress, e.g., due to handling, and pain during castration. Therefore, this study was conducted under light isoflurane anaesthesia. The level of anaesthesia was adjusted such that movement reactions to a peripheral nociceptive stimulus still occurred. We present the results of the investigation of two possible parameters for the detection of nociception: the reaction pattern in the electroencephalogram (EEG) and FOS protein expression in the spinal dorsal horn. In the electroencephalogram, a biphasic reaction pattern in response to noxious stimulation was detected that was attenuated or altered by the application of local anaesthetics. FOS expression, which was examined postmortem, was decreased after the administration of local anaesthetics, except for bupivacaine. Based on these results, local anaesthesia decreases nociceptive transmission during piglet castration in this experimental setup. When combined with the corresponding haemodynamic parameters and the evaluation of defensive movements presented elsewhere, an overall understanding of the nociceptive response to castration can be generated.

**Abstract:**

The objective of this study was to investigate the electroencephalographic reaction pattern and FOS protein expression in male piglets undergoing surgical castration under light isoflurane anaesthesia with or without local anaesthesia. The experiment was conducted under isoflurane anaesthesia to exclude the effect of the affective components of pain on the measurements. Changes in the oscillatory activity of the cerebral cortex over a 90 s period after noxious stimulation or simulated interventions were analysed. FOS expression was determined postmortem by performing immunohistochemistry in the dorsal horn of the spinal cord. The analysis of the response to an interdigital pinch revealed a biphasic reaction pattern in the electroencephalogram (EEG) that similarly was observed for the surgical stimuli during the castration procedure in the group without analgesia. This EEG response was attenuated or altered by the application of local anaesthetics. Immunohistochemical staining for FOS indicated a lower expression in the handling and in three local anaesthetic groups than in the animals castrated without pain relief. The findings indicate that EEG and FOS expression may serve as indicators for nociception in piglets under light isoflurane anaesthesia. A lower activation of nociceptive pathways occurs during castration after the application of local anaesthetics. However, EEG and FOS analyses should be combined with additional parameters to assess nociception, e.g., haemodynamic monitoring.

## 1. Introduction

Surgical castration of male piglets of less than eight days of age without anaesthesia has been a common practice for decades in several countries to prevent boar-tainted meat and aggressive behaviour of intact male pigs, as well as to obtain the requested meat quality. However, surgical castration without pain relief is a painful intervention [1,2,3]. Significant efforts have been devoted to developing methods that provide effective analgesia for the surgical castration of piglets and techniques for the examination of pain and nociception during this procedure.

Pain is a complex multidimensional experience involving sensory and affective (emotional) components that is only experienced by conscious animals and is a subjective emotion [4,5]. The present study aimed to measure nociception, the sensory component of pain, and to assess the efficacy of local anaesthetics during the castration of male piglets. Anaesthesia with isoflurane was administered to exclude the effects of affective components on the measurements. Thus, the effects of fear and stress on modulating the pain experience due to the conscious experience of handling, fixation or noises were eliminated. As isoflurane has no or negligible analgesic properties, a nociceptive response was still present. We aimed to establish a depth of anaesthesia at which the nociceptive withdrawal reflex was still present.

EEG and FOS expression were evaluated at two different locations in the nociceptive pathway: FOS expression was measured in the dorsal horn of the spinal cord, which is the first relay station in nociceptive transmission of peripheral stimuli, and pain-induced changes in cortical (electrical) activity were recorded using an EEG, representing the final stage of nociceptive signalling—the brain.

EEG, a direct measure of brain electrical activity, enables the immediate assessment of possible pain-induced changes. However, the appropriate identification of EEG changes caused by noxious stimulation is difficult due to the heterogeneity of possible EEG responses [6,7]. Numerous studies have evaluated changes in the EEGs of animals caused by noxious stimulation under general anaesthesia [8,9,10,11,12,13,14,15,16]. In piglets, EEG measurements have also been conducted to investigate the cortical responses to noxious stimulation. The results describe EEG changes following castration [17,18], exposure to other surgical stimuli [19,20,21] and innocuous noxious stimuli [22]. Some studies in animals have revealed reactions, while others failed to identify changes in EEG parameters following noxious surgical stimulation under general anaesthesia. All of these studies used processed EEG parameters, such as total power or spectral edge frequencies, derived from the EEG power spectrum to describe their findings. However, the mere use of these processed parameters complicates the ability to compare findings from different studies. Therefore, the general spectral EEG response to noxious stimulation is presented in a detailed manner as density spectral arrays, an approach that does not reduce information due to the use of processed EEG parameters.

Peripheral noxious stimulation evokes the rapid expression of the immediate early gene *c-FOS* and its protein product FOS in the nuclei of postsynaptic neurons in the spinal dorsal horn [23,24]. The expression of *c-FOS* and FOS in neurons of the spinal cord is an established marker to quantify neural activity in response to noxious stimulation postmortem [25,26]. It was applied in pigs as well, and single studies have evaluated FOS expression to investigate pain or nociception, respectively, following piglet castration [27,28,29,30,31].

The group without further analgesic treatment (NaCl) was compared with the animals that received local anaesthesia with either 0.5% bupivacaine, 2% lidocaine, 2% mepivacaine or 4% procaine, as well as with the animals that underwent only simulated interventions to correlate the observed changes in EEG patterns and FOS expression with nociception.

In this study, electroencephalographic reactions in the cerebral cortex and changes in FOS protein expression in the spinal dorsal horn of surgically castrated male piglets under light isoflurane anaesthesia are described. These measurements were evaluated as additional parameters of nociception in a multiparametric study. The corresponding results for haemodynamic and neurohumoral measurements and the evaluation of limb movements, which were investigated in the same piglets, are presented in a study by Saller et al. [3].

## 2. Materials and Methods

The study was performed in compliance with the EU Directive 2010/63/EU for animal experiments and the German Animal Welfare Act [32]. All procedures were approved by the Ethics Committee for Animal Experiments of the Government of Upper Bavaria, Munich, Germany (Reference Number ROB-55.2-2532.Vet_02-19-11).

### 2.1. Animals

Forty-nine healthy male hybrid German Landrace/German Large White × Pietrain piglets from 15 litters aged three to seven days were included in this study. Pregnant sows were sourced from a commercial piglet producer and transferred to the animal husbandry unit of the Clinic for Swine (Center for Clinical Veterinary Medicine, LMU Munich, Oberschleissheim, Germany). Sows and piglets were housed according to the German Order for the Keeping of Productive Animals and the EU Directive 2010/63/EU for animal experiments. Using a computer-generated randomised group assignment, animals were distributed into six treatment groups: four groups received either 4% procaine hydrochloride (1), 2% lidocaine hydrochloride (2), 0.5% bupivacaine hydrochloride (3) or 2% mepivacaine hydrochloride (4) as a local anaesthetic, one group received an injection of saline without further analgesia (5) and one handling group underwent only simulated interventions (6).

The study was designed as a randomised, double-blind study.

### 2.2. Experimental Protocol

Anaesthesia was induced with an initial concentration of 5% isoflurane (Isoflurane Baxter vet., Baxter Deutschland GmbH, Unterschleissheim, Germany) in 3 L/min oxygen via a face mask. Piglets breathed spontaneously during the entire trial. When the muscles relaxed and the righting reflex was no longer elicited (no reaction when the animal was placed in the supine position), the concentration of inspiratory isoflurane was reduced.

After the induction of anaesthesia, five 27-gauge disposable stainless steel needle electrodes were placed subcutaneously on the head of the piglet as follows: recording electrodes were placed supraorbitally in the area of the frontal bone on both sides of the head, two reference electrodes were placed side by side in the midline of the head at the highest point of the skull, and the ground electrode was placed in the neck. Cortical signals were recorded continuously throughout the experiment with a commercial EEG monitor (Narcotrend©, MT Monitortechnik GmbH & Co., KG, Bad Bramstedt, Germany). EEG data were recorded from two bipolar channels with a sampling rate of 128 Hz.

After placing the EEG electrodes, the piglet was turned to the supine position to insert catheters into the carotid artery and the jugular vein to monitor blood pressure and heart rate and withdraw blood samples. During all noxious interventions, the occurrence of limb movements was also recorded (a detailed description of the procedure and results has been published by Saller et al. [3]).

Following the measurement preparations, inspiratory isoflurane was further reduced to 1.5%. The appropriate concentration of isoflurane (presence of nociceptive withdrawal reflex) was evaluated in every piglet individually by applying a Pean clamp to pinch the interdigital skin fold of the hind limb. The clamp was closed maximally to the first ratchet for a maximum time of 5 s or until movement was elicited, whichever period was shortest. An anaesthetic level allowing for a slight movement reaction of the stimulated limb, provoking neither inappropriately intense movement reactions nor awakening the piglet, was determined to be appropriate. If no movement reaction occurred, the inspired isoflurane concentration was reduced in 0.2% steps and subsequently adjusted until a slight limb movement occurred. In contrast, prolonged paddling and/or movement of the forelimbs, back or head were assessed as being too excessive for the experimental setup, and the inspired isoflurane concentration was increased in steps of 0.2% and equilibrated for 3 min. This approach resulted in a mean end-tidal isoflurane concentration of 1.69 ± 0.3% for castration.

After the adequate hypnotic state was reached, an injection of either local anaesthetics or saline was performed, except in the handling group, where injections and surgical interventions were only simulated. The timeline for the overall experiment is presented by Saller et al. [3].

All piglets, except for the handling group, received a 0.5 mL intratesticular and a 0.5 mL subscrotal injection per testis. The applied local anaesthetics were 0.5% bupivacaine hydrochloride (Bupivacain 0.5%, JENAPHARM, Mibe GmbH Arzneimittel), 2% lidocaine hydrochloride (Xylocitin^®^ 2%, Mibe GmbH Arzneimittel, Brehna, Germany), 2% mepivacaine hydrochloride (Mepidor^®^ 20 mg/mL solution for injection in horses, Richter Pharma AG, Wels, Austria) and 4% procaine hydrochloride (4% procaine hydrochloride-VMD, V.M.D. sa, Arendonk, Belgium). The pH values of the four local anaesthetics ranged from 3 to 6.5 (pH values according to the manufacturer: 4% procaine hydrochloride 3–4.5, 0.5% bupivacaine 4–6, Mepidor^®^ ~5.4, and 2% Xylocitin^®^ 5.5–6.5). Two control groups were included in the study: one group that received a saline injection (0.9% NaCl, castration without pain relief) and the sham group that underwent only a simulated injection and castration (handling). An automatic self-filling 1 mL syringe (HSW ECO-MATIC^®^, Henke-Sass, Wolf GmbH, Tuttlingen, Germany) with a 25 G cannula (0.5 × 16 mm, B. Braun TravaCare GmbH, Hallbergmoos, Germany) was used for the injection. The testis was fixed with the thumb and index finger for the intratesticular injection. Subsequently, a small skin fold was formed over the testis, while the cannula was retracted until the tip was located subcutaneously, and the subscrotal injection was performed. The handling group was manipulated and fixed in the same manner as the other groups. The automatic syringe was pushed only slightly against the testes with the needle capped to simulate the injections in this group.

After an exposure time of 20 min, surgical castration was initiated (except in the handling group). In the case of castration, the skin was opened with a scalpel by creating two sagittal incisions down to the visceral layer of the vaginal tunic, and the testes were exposed. After a stabilisation period of two minutes, the testes were grasped, and the spermatic cords were successively severed with an emasculator. The blunt back of a scalpel handle was moved over the scrotum to imitate the skin incision in the animals of the handling group.

Anaesthesia was maintained for another 90 min before each piglet was euthanised with an intravenous overdose of pentobarbital (Euthadorm 500 mg/mL, CP Pharma, Burgdorf, Germany).

For the evaluation of EEG reactions, the EEG raw traces were visually inspected for artefacts or EEG burst suppression (BSupp) patterns offline. Because BSupp EEG characteristics strongly deviate from the EEG patterns present under general anaesthesia without BSupp, the decision was made to exclude these segments from further analysis.

All animals received the entire set of noxious stimuli except for the handling group, in which only interdigital pinches and simulated interventions were tested. In the piglets that underwent more than one interdigital pinch for the adjustment of anaesthetic depth, the pinch that was included in the analysis was the one at which the anaesthetic depth was evaluated to be appropriate.

Each bilateral intervention injection, skin incision and cutting of the spermatic cord was performed within a few seconds on both testes. The injection required approximately 9 s to complete per testis, and skin incisions and dissections of the spermatic cord required approximately 6 s each to complete. The EEG was analysed over a time span of 90 s following the bilaterally performed stimuli to fully assess the response to the nociceptive stimulus.

**Figure 1:**
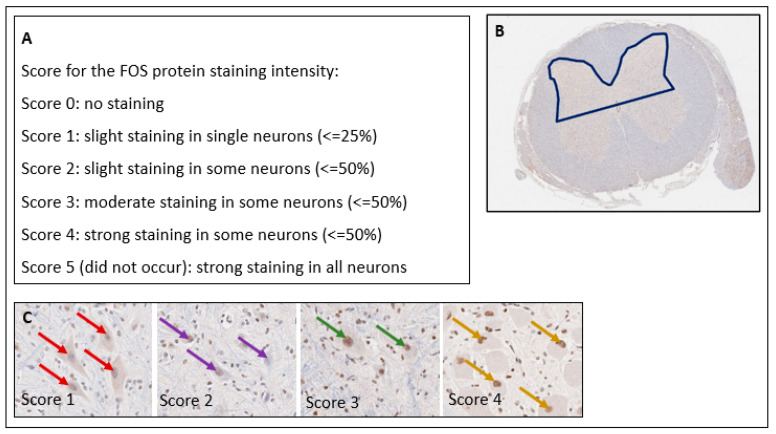
Semiquantitative Evaluation of the FOS Staining Intensity. (**A**) Score for the FOS staining intensity and frequency. (**B**) Area evaluated in the spinal cord. (**C**) Examples of different scores.

Immediately after euthanasia, lumbar and sacral segments of the spinal cord were dissected from the surrounding tissue and removed for FOS measurements. Using the vertebrae as landmarks, the spinal cord was cut into three transverse lumbar segments (L1, L2, and L3) and one sacral (S1-S3) spinal cord segment, and fixed with 38% (w/w) neutral-buffered formaldehyde for at least 48 h and embedded in paraffin (Leica ASP300S, Leica, Wetzlar, Germany). Consecutive sections (2 µm) were obtained from the cranial section of each paraffin block and stained with haematoxylin–eosin. FOS immunohistochemistry (anti-c-FOS antibody, ab209794, Abcam, Cambridge, UK, diluted in antibody diluent 1:100) was performed using a Leica Bond RXm system. Briefly, after deparaffinisation, pretreatment was performed with Epitope Retrieval 1 (corresponding to citrate buffer pH 6) for 30 min. For primary antibody binding detection and visualisation, a polymer refine detection kit was used without a postprimary antibody and with 3,3’-diaminobenzidine (DAB) as the chromogen. All slides were scanned with a Leica AT2 scanning system. A vertical line was drawn from the central canal to the lateral border of the grey matter to standardise the area for analysis. The spinal dorsal horn was defined as the grey matter dorsal to the line that was drawn (Figure 1B). The staining intensity and frequency of FOS-positive neurons in the spinal dorsal horn were evaluated semiquantitatively (Figure 1) by a German board-certified pathologist who was blinded to the groups using Aperio Imagescope Software version 12.4.0.7081.

The analysis method was first established in a preliminary study (data not shown) to develop and define a scoring approach for formalin-fixed, paraffin-embedded and stained tissue sections. The staining intensity (none, slight, moderate, or strong) and the percentage of positive neuronal nuclei in both dorsal horns of the spinal cords were calculated. A semiquantitative score including these two parameters (Figure 1A) was applied. Four different segments (L1, L2, L3, and S1-3) in each animal were evaluated. The results of this evaluation clearly showed the highest scores in the animals castrated without pain relief. Thus, it was decided to proceed with this methodological approach.

### 2.3. Statistical Analysis

#### 2.3.1. EEG

The power spectral density of the selected EEG episodes was calculated with the MATLAB pwelch function and a frequency resolution of 0.25 Hz for the pooled data from the interdigital pinch stimulus and 1 Hz for the data recorded in response to other stimuli. Because of the larger sample size in the pooled group for the pinch stimulus, we decided to use a higher frequency resolution to better describe the response. Density spectral arrays were also constructed, and the results are presented as heatmaps to depict the temporal evolution of the spectral EEG features. The DSA was derived from 5-s EEG episodes overlapping by 4 s. The results are presented as changes in the EEG power relative to a prestimulus baseline defined as the episode 50–40 s before the stimulus. The poststimulus value was divided by the prestimulus value. Thus, a value <1 indicates a higher value before the stimulus, whereas a value >1 indicates an increase after stimulation, and a value of 1 indicates no change after the stimulus. This approach helps to track stimulus-induced EEG changes and correct for different EEG amplitudes between the animals.

For the DSA plots, a signed rank test was performed for each pixel by assessing the power of the respective frequency at the observed time versus the power of the frequency at baseline. Pixels that either had a *p* < 0.05 (pooled response to the interdigital pinch) or *p* < 0.1 (single experimental group, all other stimuli) are shown.

Hence, only significant changes that occurred in clusters are discussed. The cluster approach was used because of the low probability of the concentration of spurious false positives in a cluster. Therefore, the strategy was used to define a cluster size [33], i.e., either a 4 s × 2 Hz cluster for the pooled reaction to the interdigital pinch stimulus or a 4 s × 4 Hz cluster for the reaction to other stimuli. The area under the receiver operating curve (AUC) was calculated with 10k-fold bootstrapped 95% confidence intervals to track the changes in the EEG band power over time. If the 95% confidence intervals did not cross the 0.5 line, this result was considered to be significant as long as it was observed in at least at two neighbouring time points. Similar approaches have been used previously [34,35].

MATLAB R2017a (The MathWorks, Inc., Natick, MA, United States) was used for the statistical analysis. For the AUC calculation, additional functions from the measures of the effect size toolbox were used [36]. Different settings were used for the interdigital pinch stimulus and the other noxious stimuli, as a much larger number of samples was available for the interdigital pinch by pooling data from all animals, independent of the treatment groups. Beeswarm plots were generated with the MATLAB plotSpread function.

#### 2.3.2. FOS Protein

Four tissue sections per animal were included in the analysis: one section each from spinal cord segments L1, L2, L3 and S1-3. The median staining intensity and frequency of the dark-brown DAB precipitate in the four spinal cord segments were calculated for every animal, and a Kruskal–Wallis test was applied to identify significant differences between the NaCl group and the other experimental groups. Figure 1 shows the scoring scheme applied for the FOS analysis.

## 3. Results

### 3.1. Demographics

For the EEG analysis, 196 datasets (four per animal) from 49 piglets aged 5.3 ± 1.1 days and weighing 2.14 kg ± 0.45 kg were acquired. Some animals exhibited timespans with BSupp in measurement periods; therefore, 30 datasets were discarded (27 due to BSupp presence and 3 due to artefacts), and 166 datasets were included in the analysis. The interdigital pinch was performed under isoflurane anaesthesia in the absence of any analgesic. It was applied to all animals using an identical technique. Therefore, the animals from all experimental groups were combined to evaluate the EEG changes due to the interdigital pinch (*n* = 44 datasets). For these reasons, varying numbers of datasets were analysed for the six experimental groups (Table 1).

For the FOS analysis, tissue sections from 42 animals were evaluated. Due to sampling and trimming inaccuracies, the individual spinal cord sections from seven animals could not be evaluated. Therefore, those seven animals were excluded from the final analysis. The analysed animals were 5.5 ± 1.0 days of age and weighed 2.13 kg ± 0.45 kg (Table 2).

### 3.2. Interdigital Pinch

**Figure 2:**
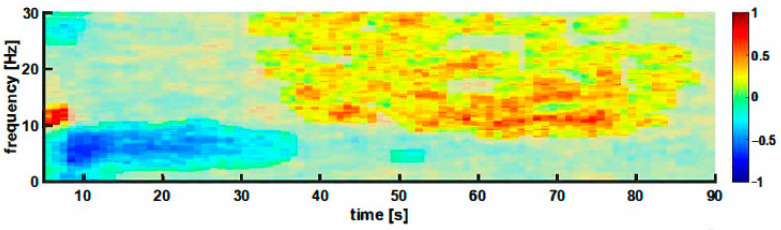
DSA of the Pooled Median Response (*n* = 44) to Interdigital Pinch Stimulation over 90 s. Areas with bold colours indicate the frequency and time regions with significant differences between pre- and poststimulus conditions. The colour bar indicates the change in the poststimulus power compared with the prestimulus baseline. The stimulus started at t = 0. Briefly, the interdigital pinch leads to an ‘early‘ reduction in power in the slow frequencies below 10 Hz and a ‘late‘ activation of higher frequencies above 10 Hz starting approximately 35 s after the stimulation.

For the pooled data (*n* = 44), a significant increase in frequencies of ~10–14 Hz was observed immediately after the stimulus was applied, along with a significant decrease in the ~0.5–8 Hz band power (delta and theta range, ‘early’ response). The decrease in power lasted until approximately 35 s after the beginning of stimulation (t = 0). After this decrease in power, an increase in power at frequencies >10 Hz was observed starting after approximately 35 s until approximately 90 s after t = 0 (‘late‘ response). Figure 2 presents the DSA of the change in power relative to the baseline and highlights the early and late components observed following the interdigital pinch.

### 3.3. Injections

The reactions to the injection stimuli were quite heterogeneous among the substances. For the NaCl, bupivacaine and procaine groups, a significant reaction was observed as an activation of frequencies of ~10–15 Hz at approximately 30–40 s after the stimulus. In particular, in the procaine group, a significant ongoing depression of ~10–15 Hz oscillations appeared starting approximately 60 s after the stimulus. For the mepivacaine and procaine groups, a reduction in power in the lower frequencies immediately after the injection stimulus was detected. Lidocaine and bupivacaine produced short, significant decreases at approximately 80 s, at frequencies of ~10–15 Hz.

Supplemental Figure S1 presents the DSAs of the changes in power compared with prestimulus conditions for the 90 s period after the intratesticular and subscrotal injections (Figure S1).

### 3.4. Skin Incision

**Figure 3:**
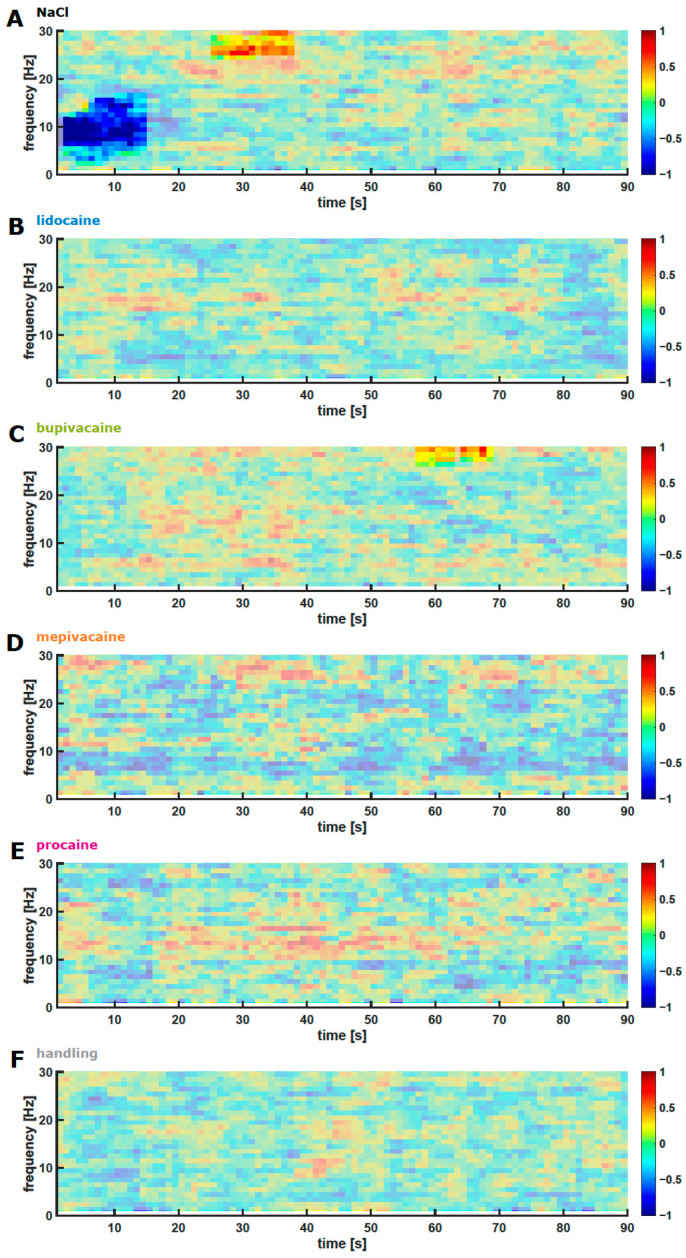
DSAs of the Changes in EEGs after the Scrotal Skin Incision. The DSAs present changes in EEG power over 90 s following the incision stimulus in the following experimental groups: NaCl (**A**), lidocaine (**B**), bupivacaine (**C**), mepivacaine (**D**), procaine (**E**), and no incision and handling only (**F**). The incision started at t = 0. Areas shown in bold colours indicate frequency and time regions with a significant difference from the prestimulus EEG. (**A**) NaCl caused an ‘early‘ and ‘late‘ response to the incision stimulus. The early response was an attenuation in the 5–15 Hz range within the first 20 s after stimulation, and the late response was an activation in the 20–30 Hz power range at approximately 30–40 s after the stimulation. (**B**–**F**) Significant changes in the EEG power were not observed in the local anaesthetic and handling groups following the stimulus, except for a high-frequency activation at approximately 30 Hz and approximately 60 s after stimulation in the bupivacaine group (**C**).

A biphasic, early and late response to the scrotal skin incision was observed in the NaCl group with an ‘early’ (~10 s) decrease of ~5–15 Hz power followed by a ’late’ activation of ~20–30 Hz power, starting at approximately 25 s. For all other groups, a significant, stimulus-induced change was not observed. Figure 3 presents the DSAs of the change in power when compared with the prestimulus conditions for the 90 s period after the incision.

### 3.5. Cutting of the Spermatic Cord

**Figure 4:**
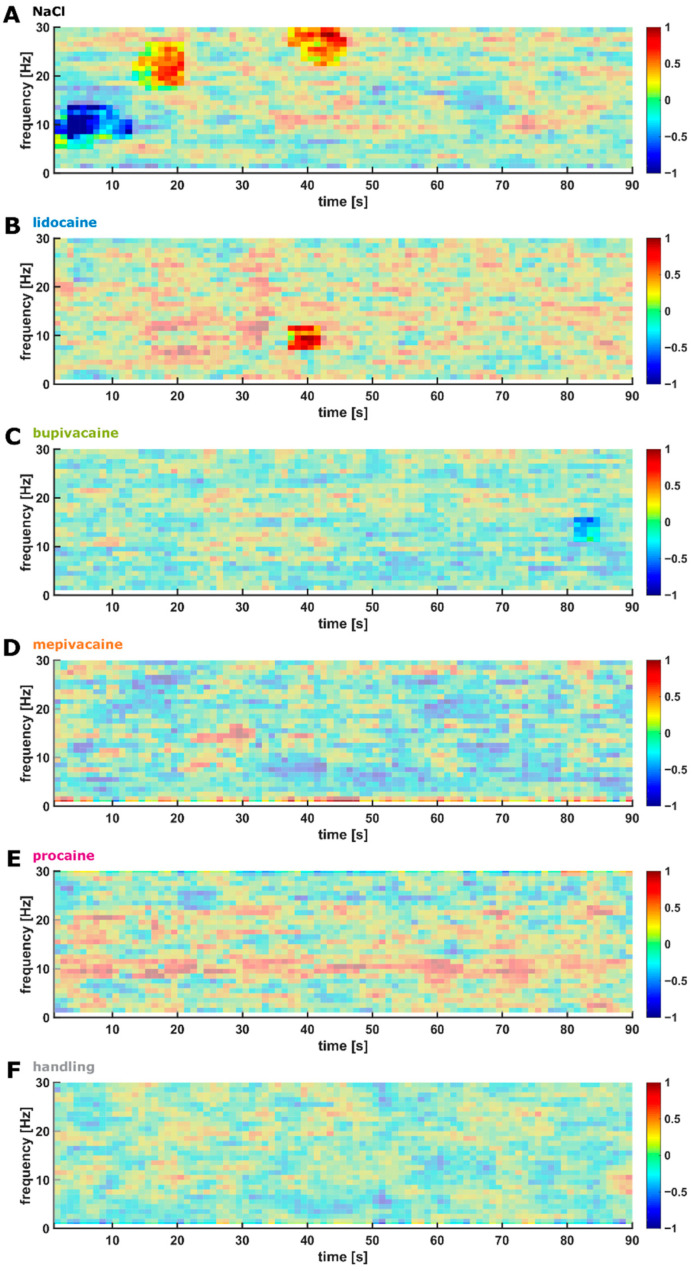
DSAs of the EEG Changes after Spermatic Cord Dissection. The DSAs present changes in EEG power over 90 s following spermatic cord dissection in the following experimental groups: NaCl (**A**), lidocaine (**B**), bupivacaine (**C**), mepivacaine (**D**), procaine (**E**), and no incision or handling only (**F**). Stimulus started at t = 0. (**A**) In the NaCl group, an ‘early‘ and ‘late‘ response to the dissection stimulus was observed. The early response was an attenuation in the ~5–15 Hz range within the first 15 s after stimulation, and the late response was an activation of ~20–30 Hz at approximately 20 and 30–40 s after stimulation. (**B**–**F**) No significant changes in the EEG power were observed in the local anaesthetic and handling groups following the stimulus, except for an activation of ~10 Hz power at approximately 40 s after stimulation in the lidocaine group (**B**) and a late decrease in ~15 Hz power at approximately 80 s in the bupivacaine group (**C**).

Analogous to the incision and the interdigital pinch, a biphasic response to spermatic cord dissection was detected in the NaCl group, with an early (~10 s) decrease in ~5–15 Hz power followed by a late activation of ~20–30 Hz power starting at approximately 20 s. This biphasic response was not observed in any of the other groups. For the lidocaine group, an increase in ~10 Hz power was observed approximately 40 s after noxious stimulation. For all other groups, no specific reaction to the stimulus was detected. Figure 4 presents the DSA of the change in power for the 90 s period after spermatic cord dissection, compared with the prestimulus conditions.

### 3.6. Evaluation of the Early and Late Responses

**Figure 5:**
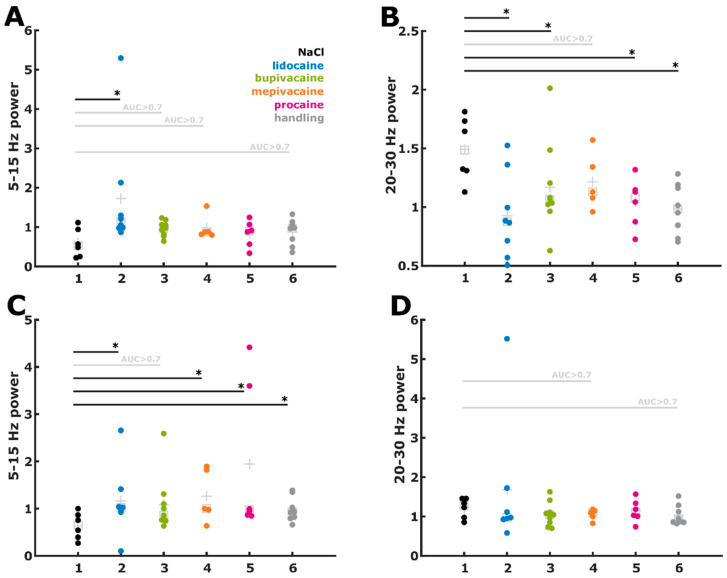
Comparison of ‘early‘ and ‘late‘ reactions following skin incision (**A**,**B**) and spermatic cord dissection (**C**,**D**), compared with the NaCl group. (**A**) For skin incisions, a significant (*) or possibly relevant (AUC > 0.7) effect was observed for the early response in all groups, except for the procaine group. The 5–15 Hz power decreased by a smaller amount as a reaction to the stimulus when compared with the change in the NaCl group.(**B**) Regarding the late response, all groups showed a weaker activation of 20–30 Hz power as a result of the stimulation compared with that of the NaCl group. (**C**) As an early reaction following spermatic cord dissection, all groups exhibited a weaker 5–15 Hz response than the NaCl group. (**D**) Significant differences in the late 20–30 Hz power responses were not observed after spermatic cord dissection.

The NaCl group showed a similar reaction pattern following skin incision and cutting of the spermatic cord, namely, an early decrease in low-frequency waves followed by an increase in high-frequency waves (late response). The detection periods of all local anaesthetic groups and the handling group were compared with those of the NaCl control. Figure 5 presents beeswarm plots of the statistical analyses of the early (5–10 s after stimulus) decrease in the frequency bands of 5–15 Hz and the late (30–40 s after stimulus) increase in the frequency bands of 20–30 Hz. The reactions of the different substance groups and the handling group to skin incisions (Figure 5A,B) and spermatic cord dissections (Figure 5C,D) were compared with the NaCl group.

### 3.7. FOS Protein

**Figure 6:**
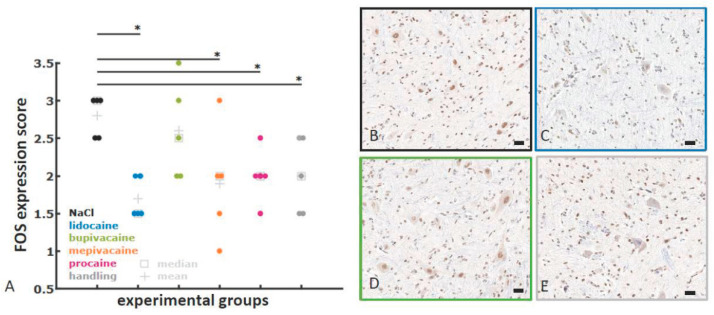
Scatter plot of the FOS staining score (**A**) and representative images of FOS protein expression (**B**–**E**). (**A**) Significantly (AUC 95% CI except 0.5) less FOS protein expression was observed in the handling group and in all local anaesthesia groups, except for the bupivacaine group, than in the NaCl group. (*) A significant effect (*p* < 0.05). (**B**–**E**) Representative images of FOS protein expression in the dorsal horn of the spinal cord in animals from the NaCl (**B**), lidocaine (**C**), bupivacaine (**D**), and handling (**E**) groups (bars indicate 30 µm).

Significantly lower expression of the FOS protein was observed in the analysed lumbar (L1, L2, L3) and sacral (S 1-3) segments of the spinal dorsal horn in the handling group and in all local anaesthetic groups, except the bupivacaine group, when compared with the NaCl group. All animals, including the handling group, displayed at least slight staining in <25% of the neurons that resulted in a minimum score of one. Figure 6 displays the corresponding scatter plot as well as images of the immunohistochemical staining.

Additionally, FOS protein expression was evaluated only in animals that showed no BSupp patterns throughout the measured EEG epochs (Figure S2).

## 4. Discussion

The initial analysis of the changes in EEG patterns following the interdigital skin fold pinch, which induced a nociceptive withdrawal reflex, showed a strong biphasic response. As this stimulus was applied to all animals in an identical manner without analgesia, the data were pooled. This approach yielded a large number of samples. The biphasic EEG response consisted of an ‘early’ and a ‘late’ component. Similar EEG reactions were recorded following the scrotal skin incision and dissection of the spermatic cord without local anaesthetics. The application of local anaesthetics attenuated cortical reactions. Additionally, FOS protein expression in the dorsal horn of the spinal cord was reduced by most local anaesthetics.

Using an interdigital pinch, which was reported to be a strong noxious stimulus in newborns and juvenile pigs [37,38], the adequate anaesthetic level for each animal was determined individually. The presence of a nociceptive withdrawal reflex induced by interdigital pinching is a well-accepted indicator of nociception during anaesthesia in various species and in pigs [39,40,41].

BSupp, a pattern of waxing and waning amplitudes, may reflect a state of excessively deep anaesthesia, but it may also occur at low anaesthetic doses in the developing pig [42,43]. In the present study, some piglets showed time spans with BSupp, and thus they were excluded from the analysis. In a previous study of juvenile pigs, individual animals also exhibited BSupp at isoflurane concentrations of sub-MAC levels [44]. In general, isoflurane depresses the dose-related cortical electrical activity in juvenile pigs [42]. However, the occurrence of movement reactions to noxious stimulation in juvenile pigs and rats with BSupp has been reported [37,45,46].

### 4.1. EEG Reaction

Piglet castration includes two distinct painful aspects, incision of the scrotal skin and pulling and cutting of the spermatic cords, which is suggested to be the most painful component of castration [3,47]. In the EEG measurements, both noxious stimuli were analysed separately. The cortical reactions in the NaCl group were expected to reflect the unaltered and most intense noxious stimulus in this experiment because no analgesia was applied. For this group, a biphasic response to both the skin incision and cutting of the spermatic cords was observed that was similar in shape to the interdigital pinch response. The EEG reaction was altered or strongly attenuated in the local anaesthetic and handling groups. After a prior application of either lidocaine, mepivacaine or procaine, the incision of the scrotal skin evoked no significant reactions in the cerebral cortex, or at least a strongly attenuated reaction, in the bupivacaine group. Following the cutting of the spermatic cords, a short but significant activation was observed in the lidocaine group, but no significant changes were detected in the other local anaesthetic groups. The comparison of the intense changes during the early and late response observed in the NaCl group with that in the groups that received local anaesthetic and the handling group also suggests weaker responses in the local anaesthetic groups and in the handling group.

The EEG reaction to noxious stimulation under general anaesthesia may be manifold [6]. In patients under general anaesthesia, noxious stimulation may decrease EEG power in the alpha band [48,49] or increase the power in the delta band [50] or beta band [51]. Various EEG reactions to noxious stimulation that may depend on the species, age, the type and dose of general anaesthetic used, the type and intensity of stimulation and the amount of analgesic have also been reported for interventions in animals [8,11,12,44,52,53].

The observed EEG reaction to the interdigital pinch with an early decrease in slow oscillatory activity followed by an increase in oscillatory activity above 10 Hz most likely indicates a change towards arousal. The additional strong increase in power in the 10–15 Hz range during the first seconds after the stimulus began may be attributed to the clamp that was still attached to the skin fold. EEG arousal indicates a shift in EEG activity from the lower to the higher frequency range. These higher frequencies arise from a desynchronisation of neuronal activity and are usually associated with higher cortical function [54]. The arousal reaction may be regarded as a change in the EEG pattern in the direction of the conscious EEG pattern [17]. It is not a specific indicator of nociceptive input but represents a common reaction to noxious stimulation in humans and has also been described in animals [9,14,18].

Haga et al. [22] did not observe significant EEG changes in developing pigs following innocuous noxious mechanical stimulation under isoflurane anaesthesia. An underlying biphasic response pattern as observed in the present study may have masked the findings. Other findings seem to be similar to the present results. EEG data recorded in adult goats and averaged over 1 min showed that the goats reacted to a clamp on the dew claw under isoflurane anaesthesia with decreases in the processed parameters total, delta, theta and alpha power, while the beta power was unchanged [10]. Nevertheless, the results may not be directly comparable with those of the present study due to differences in several parameters, e.g., species, age, EEG segment length used for analysis and the use of processed EEG parameters.

EEG measures were also used to examine surgical noxious stimulation induced by tail docking in piglets. A comparison of EEG responses in piglets of either 2 or 20 days of age showed an effect of age on the EEG reaction [19]. Young piglets showed little EEG response to tail docking, whereas older piglets reacted with significant changes in median frequency and total power. Johnson et al. also detected different EEG responses in piglets between 1 and 15 days of age [21]. The varying EEG reactions of piglets in the first 20 days of life imply that major changes occur in the EEG responses of piglets to noxious stimulation, particularly during the first weeks of life. Both studies documented changes in processed EEG parameters following tail docking under halothane anaesthesia, but detailed EEG data are missing. Different noxious stimuli cause differing EEG changes as well [12]. Therefore, a comparison with the present results is only possible under restrictions.

Studies of EEG responses to surgical castration in piglets exist, although none of these studies were performed under isoflurane anaesthesia. However, the anaesthetic agent used is crucial because different anaesthetic agents induce different EEG patterns [34,53,55,56], and the choice of anaesthetic agent may partially account for differences in results between EEG patterns in nociceptive studies [7]. Initial findings from Waldmann et al. [18] on the castration of piglets also reveal EEG arousal reactions in the EEGs of individual piglets following castration under barbiturate anaesthesia. Haga and Ranheim [17] observed decreases in delta, theta, and alpha bands and total EEG power following castration under halothane anaesthesia without additional analgesia in approximately three-week-old piglets. Piglets that received lidocaine either intrafunicularly or intratesticularly showed a less pronounced EEG response [17]. The presented results tend to be similar, as reactions in the cerebral cortex were decreased or at least altered after local anaesthesia was applied. Because of the use of EEG band power and the lack of detailed information regarding changes in the EEG spectrum in these studies, comparing the detailed EEG responses is not possible.

The injection of local anaesthetics yielded the expected heterogeneous EEG reactions. The data indicate that intratesticular and subscrotal injections provoke significant changes in EEG waves, irrespective of which fluid is used. The reactions differed in all groups in regards to the biphasic reaction pattern observed following the interdigital pinch, or noxious stimulation by skin incision and dissection of the spermatic cord when no local anaesthetics were coapplied. The varying pharmacological properties of the local anaesthetics may cause different stimuli that are not uniform and might explain the various postinjection EEG patterns. Nonetheless, the datasets from the injections are considered valuable and are presented as supplemental data (Figure S1).

For the presentation of the EEG results, the power spectrum used for the generation of the DSA plots from 5 s EEG episodes with a 1 s overlap was calculated with the goal of not missing any EEG reactions with a short duration. Although the *p* < 0.1 threshold is unusual, it may help us better identify the possible response; however, due to the limited sample size, the findings from the different groups are preliminary results. The study refrains from discussing scattered pixels because of the increased risk of discussing false positives due to multiple comparisons, which is why the cluster approach was used.

We refrained from focusing on changes in predefined frequency bands or in parameters using information from the entire spectrum. First, the division of the EEG power spectrum of animals into the frequency bands alpha, beta, delta and theta may be quite arbitrary [7], and variability has been observed in the specific frequency range that defines each band [57]. Second, processed parameters, such as the median frequency or wide frequency bands (e.g., the beta range from ~12–30 Hz), might mask effects. For instance, an increase in power from 12–16 Hz and a simultaneous decrease in power from 20–25 Hz would not have an effect on the beta band.

In general, differences in the anaesthetic agent used, noxious stimuli, species, age of the animals, evaluated parameters, and time scales prevent a comparison of studies. This study presents the unprocessed spectral response in the EEG, which may facilitate the translation of the findings to the results from other experiments. The use of DSA plots and the decision to refrain from using processed EEG parameters allowed us to observe chronological changes in EEG activity patterns over the entire frequency range. This approach describes the EEG reactions in a more detailed manner than using processed EEG parameters or band powers derived from the spectrum.

### 4.2. FOS Protein

The expression of the immediate early gene *c-FOS* and its protein product FOS in neurons of the spinal cord is a well-established marker to quantify neural activity in response to noxious stimulation [25,26]. Noxious peripheral stimulation evokes a rapid change in gene expression of the postsynaptic neurons in the dorsal horn of the spinal cord [24]. The mRNA of the immediate early gene *c-FOS* is detected within five minutes following physical stress, such as injury [58]. The *c-FOS* gene encodes the nuclear protein FOS, which is detected in the same neurons with a protein peak observed at approximately 90–120 min after the induction of gene expression [23,25,59]. After noxious peripheral stimulation, immunohistochemical FOS-positive neurons are predominantly observed in laminae I and II of the dorsal horn, along with some labelling in laminae III-VI [26,27]. A semiquantitative approach was used to evaluate FOS expression in the spinal dorsal horn, which includes laminae I–VI. This approach has not been validated for use as a stand-alone read-out of neuronal stimulation in the spinal cord. However, in combination with the EEG results, it serves as an additional parameter, which can be used to further evaluate the nociceptive response.

The majority of afferent neurons supplying the porcine testis and adjacent structures are located in dorsal root ganglia of lumbar segments one to three and sacral segments one to three [60,61,62]. Therefore, these spinal cord segments were included in the analysis. Significantly lower levels of FOS expression were detected in the nuclei of spinal neurons in the piglets that were castrated after application of a local anaesthetic, except for the bupivacaine group, and in animals that were only handled, compared with animals undergoing castration without analgesia. FOS expression was additionally analysed in only the animals that did not show BSupp throughout the experiment to evaluate a possible effect of the anaesthetic level (i.e., BSupp). The results were similar and are presented in the supporting information (Table S2). The FOS results support data from a study by Nyborg et al., who reported a lower number of FOS-positive neurons in the spinal dorsal horn in piglets undergoing castration with local anaesthesia than in piglets undergoing surgery without analgesia [29]. FOS expression in the dorsal horn of piglets was also reduced after castration under CO_2_ anaesthesia [28]. Additionally, local anaesthesia was effective at reducing the number of FOS-positive neurons in juvenile pigs undergoing laparotomy when compared with animals undergoing surgery without anaesthesia [27].

In the present experiment, all animals showed at least slight staining in single neurons. This finding may result from the fact that FOS expression in this experiment represents the cumulative response to noxious stimulation occurring in the time period of ~90–120 min before harvesting the spinal cord and transferred via neurons in L1, L2, L3 and S1-3. All animals experienced at least two interdigital pinches, the second of which was applied four minutes following spermatic cord dissection and therefore occurred in the relevant time frame. Due to the similar innervation of the interdigital area of the hind claw, the interdigital pinches very likely contributed to FOS expression in the present study. Moreover, changes in heart rate and mean arterial blood pressure in the handling group were observed following injection, skin incision and spermatic cord dissection [3]. Obviously, the touching and fixation of the testes also led to adverse reactions, which possibly also contributed to the baseline FOS expression. Despite baseline FOS expression, significant differences in FOS expression were evident between the animals treated without analgesia and animals treated with local anaesthesia or only-handled animals. Therefore, we assumed that FOS expression may be used as an indicator of nociception during suckling piglet castration. However, further studies with larger animal numbers would be beneficial to confirm this finding.

One limitation of the study is that a sample size calculation for the overall study was not performed based on EEG effects or FOS expression. Haga, Tevik and Moerch [22] found blood pressure to be a more sensitive indicator of nociception in juvenile pigs than electroencephalographic parameters. Therefore, the sample size estimation was specifically based on the corresponding haemodynamic parameters. Hence, the power of the present study may be too low to draw definite conclusions based on EEG and FOS measurements as stand-alone parameters regarding the efficacy of the four local anaesthetics for piglet castration or to compare them with each other. Further studies with a larger sample size are needed to describe the effects on the local anaesthetic groups in more detail and to compare the groups.

## 5. Conclusions

The EEG reactions of 3- to 7-day-old piglets to an innocuous interdigital pinch and to surgical castration under light isoflurane anaesthesia were tracked. Both the noxious stimulation caused by an interdigital pinch and by castration without pain relief were visible in the EEG as a biphasic ‘early’ and ’late’ response. This EEG reaction pattern was attenuated in the animals that received local anaesthesia and in animals of the handling group. We are confident in our identification of the nociceptive response in the EEG because of the (i) repetitive nature of the signals observed following interdigital pinching and surgical interventions without analgesia, (ii) the attenuated response to surgical intervention with local anaesthetics, and (iii) the reduced FOS expression in animals treated with local anaesthetics.

It can be assumed that the use of local anaesthetics in piglets undergoing surgical castration under isoflurane anaesthesia very likely attenuates nociceptive transmission under standardised laboratory conditions.

The EEG and FOS results are consistent with the significantly reduced heart rate and blood pressure and the decreased movement reactions that were detected in the same animals [3]. The results of the EEG and FOS measurements in combination with the corresponding results for the haemodynamic parameters and the evaluation of limb movements [3] provide valuable information with which to form an overall understanding of the nociceptive response of piglets to castration with or without local anaesthetics.

Additionally, the results from the EEG and FOS analyses provide strong pilot data for further studies regarding EEG nociception in piglets. To our knowledge, the detailed EEG reactions of piglets to a noxious interdigital pinch and surgical castration, as well as a description of how local anaesthetics may alter this pattern, have not been presented to date. Further investigations are required to determine whether or how the results relate to pain perception in conscious piglets and under field study conditions.

## Figures and Tables

**Table 1:** Overview: Number of Datasets Used in the EEG Analysis.

Experimental Group	Noxious Stimulus			
	Interdigital Pinch	Injection	Incision	Cutting of the Spermatic Cord
NaCl (*n* = 8 animals)	*n* = 44 datasets (pooled data)	*n* = 7 datasets	*n* = 6 datasets	*n* = 6 datasets
Lidocaine (*n* = 9 animals)		*n* = 7 datasets	*n* = 8 datasets	*n* = 7 datasets
Bupivacaine (*n* = 9 animals)		*n* = 9 datasets	*n* = 9 datasets	*n* = 9 datasets
Mepivacaine (*n* = 6 animals)		*n* = 5 datasets	*n* = 5 datasets	*n* = 5 datasets
Procaine (*n* = 8 animals)		*n* = 6 datasets	*n* = 5 datasets	*n* = 5 datasets
Handling (*n* = 9 animals)		*n* = 7 datasets	*n* = 8 datasets	*n* = 8 datasets
Total *n* = 49 animals	Total *n* = 166 datasets			

**Table 2:** Overview: Number of Animals for the FOS Analysis.

FOS protein Analysis	NaCl	Lidocaine	Bupivacaine	Mepivacaine	Procaine	Handling
(*n* = 42 animals)	*n* = 7	*n* = 7	*n* = 8	*n* = 7	*n* = 6	*n* = 7

## Data Availability

Not applicable.

## Supplementary Materials

The following supporting information can be downloaded at https://www.mdpi.com/article/10.3390/ani12182309/s1, Supplementary Material 1: Figure S1: EEG Changes after Intratesticular and Subscrotal Injections; Supplementary material 2: FOS Protein Analysis in Only Animals without BSupp; Figure S2: Scatter Plot of the FOS Staining Score (Based on Animals without BSupp); and Table S2: Number of Animals Included in the FOS Protein Analysis (Based on Animals without BSupp).
