# Supplementary material for: Nociception-Induced Changes in Electroencephalographic Activity and FOS Protein Expression in Piglets Undergoing Castration under Isoflurane Anaesthesia
Source: Animals (Basel). 2022 Sep 6;12(18):2309. doi: 10.3390/ani12182309 (PMC9494976; doi:10.3390/ani12182309)
Supplement: Supplementary file 1 [file animals-12-02309-s001.zip › Supplementary file 1.pdf]

Supplementary file 1

EEG Changes After Intratesticular and Subscrotal Injection

Regarding intratesticular and subscrotal injection, common features of all treatment groups were the standardized injection technique and volume. However, the pharmacokinetic and physical-chemical properties differ between the four local anesthetics (e.g., amid- or ester-type, anesthetic potency, lipid solubility, pH and pKa values, and protein binding) and saline. This variance in properties of the local anesthetics indicates that the stimulus was not uniform and may have caused the various EEG patterns induced by the different substances post-injection.

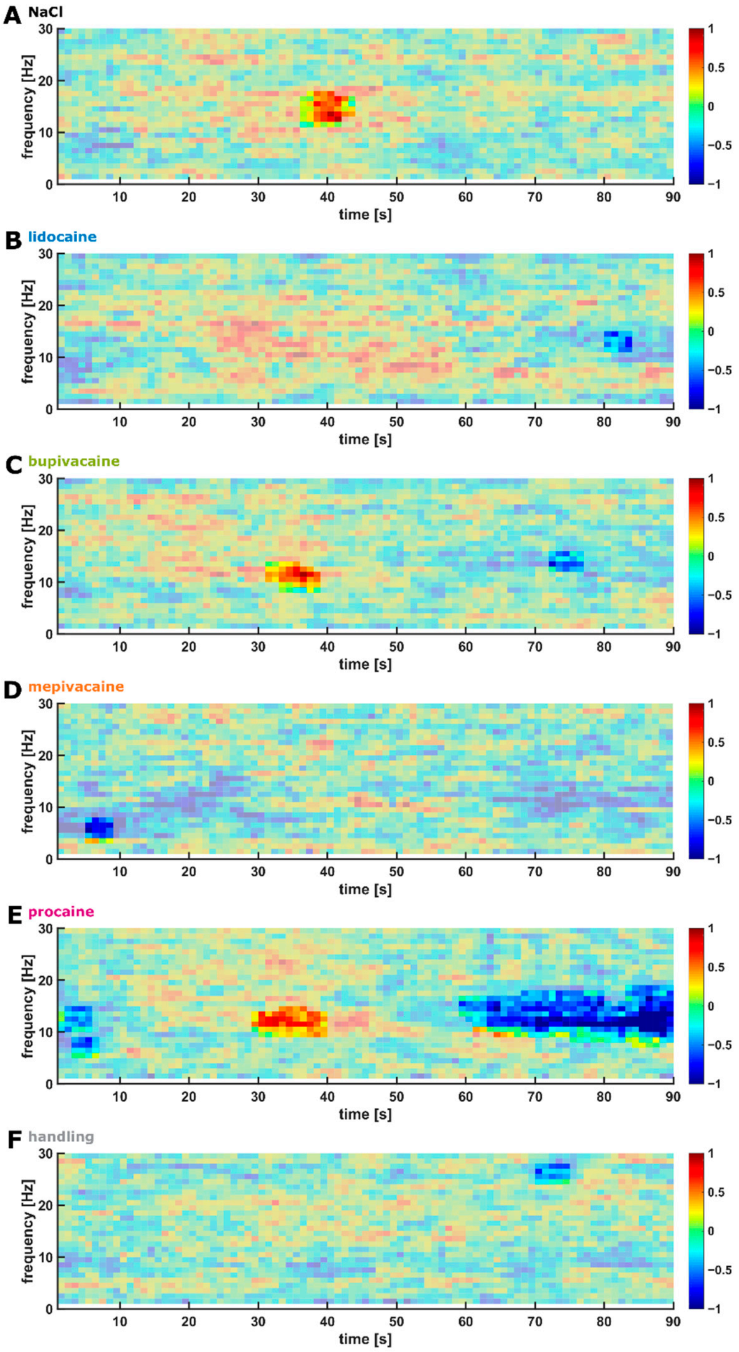

Figure S1. DSAs of the EEG Changes After Intratesticular and Subscrotal Injection. The DSA plots present changes in EEG power after injection of NaCl (A), lidocaine (B), bupivacaine (C), mepivacaine (D), or procaine (E), as well as no injection and only handling (F). Injection started at t=0. Areas with bold colors indicate frequency and time regions with a significant difference from the prestimulus EEG.

A) NaCl injection led to a significant activation of frequencies ~10–15 Hz in the 30–40 s after the injection stimulus

B) Lidocaine injection caused a short significant decrease in ~10-15 Hz power at approximately 80 s.

C) Bupivacaine injection caused a significant activation of frequencies of ~10–15 Hz approximately 30–40 s after the injection and a short significant decrease in ~10-15 Hz power at approximately 80 s.

D) Mepivacaine injection triggered a significant early decrease in ~5–10 Hz power.

E) Procaine injection led to a significant activation of frequencies of ~10–15 Hz at 30–40 s after the injection stimulus.

F) In the handling group, a decrease in high-frequency power of approximately 25 Hz was observed.
