# Supplementary material for: Nociception-Induced Changes in Electroencephalographic Activity and FOS Protein Expression in Piglets Undergoing Castration under Isoflurane Anaesthesia
Source: Animals (Basel). 2022 Sep 6;12(18):2309. doi: 10.3390/ani12182309 (PMC9494976; doi:10.3390/ani12182309)
Supplement: Supplementary file 1 [file animals-12-02309-s001.zip › Supplementary file 2.pdf]

Supplementary file 2

FOS analysis in only animals without BSupp

For FOS protein detection in only animals that showed no BSupp throughout the measuring epochs, tissue sections of 34 animals could be included in the analysis. Animals were 5.4 ± 1.0 days of age, weighing 2.15 ± 0.46 kg (Table S2).

Table S2. Overview: Number of Animals Included in the FOS Protein Analysis (Based on Animals without BSupp)

**FOS protein analysis**	**NaCl**	**Lidocaine**	**Bupivacaine**	**Mepivacaine**	**Procaine**	**Handling**
**Number of animals (n=34)**	n=7	n=6	n=8	n=5	n=4	n=4

The results were similar to those presented in the analysis including all of the animals. Additionally, there was significantly (AUC 95% CI except 0.5) less FOS expression in the handling group and in the lidocaine and procaine groups than in the NaCl group (Figure S2). In the mepivacaine group, no significant but clinically relevant effect (AUC>0.7) was demonstrated. In the bupivacaine group, no significant difference compared with the NaCl group was detected.

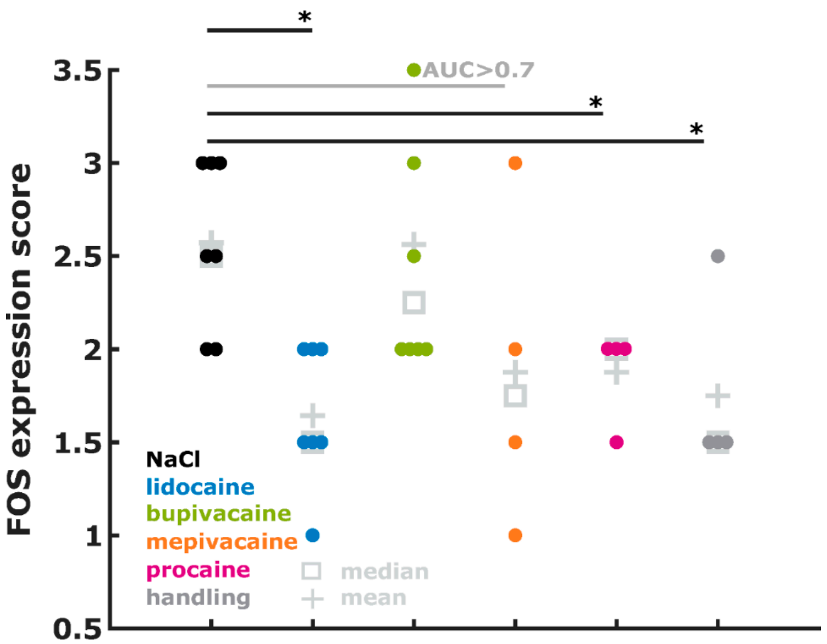

Figure S2. Scatter Plot of the FOS Expression Score (Based on Animals without BSupp).
